# Addressing challenges in information-provision: a qualitative study among oncologists and women with advanced breast cancer

**DOI:** 10.1186/s12904-021-00836-w

**Published:** 2021-09-14

**Authors:** Liesbeth M. van Vliet, Maartje C. Meijers, Sandra van Dulmen, Elsken van der Wall, Nicole Plum, Jacqueline Stouthard, Anneke L. Francke

**Affiliations:** 1 Health, Medical and Neuropsychology Unit, Institute of Psychology, Leiden University, Wassenaarseweg 52, 2333 AK Leiden, the Netherlands; 2 Leiden Institute for Brain and Cognition (LIBC), Leiden University, Leiden, the Netherlands; 3 Nivel (Netherlands Institute for Health Services Research), Utrecht, the Netherlands; 4 Department of Primary and Community Care, Radboud University Medical Center, Radboud Institute for Health Sciences, Nijmegen, the Netherlands; 5 Faculty of Health and Social Sciences, University of South-Eastern Norway, Drammen, Norway; 6 Department of Medical Oncology, University Medical Center Utrecht, Utrecht University, Utrecht, the Netherlands; 7 Netherlands Cancer Institute, Amsterdam, the Netherlands; 8 Amsterdam Public Health Institute, Vrije Universiteit, Amsterdam, the Netherlands

**Keywords:** Communication, Incurable cancer, Qualitative research, Breast cancer, Information-provision, Empathy

## Abstract

**Background:**

There is a need for more insight into how to address challenges of information-provision for women with advanced breast cancer. We aimed to explore oncologists’ and patients’ views on (i) the challenges of information-provision, and (ii) possible strategies to address these challenges, meanwhile (iii) exploring the possible facilitating role of positive expectations and empathy.

**Methods:**

Semi-structured interviews were held with oncologists (n = 10) and women with advanced breast cancer (n = 14). Principles of Thematic Analysis were followed, with two researchers analyzing transcribed data, supported by Atlas.ti software.

**Results:**

Taken together the data from oncologists and patients, we found that when communicating with patients with advanced cancer, oncologists face challenges, including handling patients’ unrealistic disease (status) beliefs, and choosing approaches for discussing available treatment options and their side effects. Possible strategies to address these challenges include balancing information with acceptance of denial, and using medical expertise to guide treatment discussions. A sensitive issue is whether to discuss the option of no anti-cancer treatment. Meanwhile, approaches and preferences for discussions of side effects vary. Positive expectations and empathy can facilitate information-provision by creating space and helping patients to open up more.

**Conclusions:**

Integrating oncologists’ and patients’ views, oncologists can provide realistic information while also, temporarily, accepting denial, and can use their medical expertise to address challenges around unrealistic beliefs and discussion of treatment options. Finding ways to tailor discussions of no anti-cancer treatment and side-effect information are needed. Positive expectations and empathy might facilitate – tailored – information-provision, leading ultimately to patient-centered care lying at the heart of medicine.

**Supplementary Information:**

The online version contains supplementary material available at 10.1186/s12904-021-00836-w.

## Introduction

Good information-provision is fundamental to patient-centered decisions and care, especially when being faced with an advanced cancer diagnosis [1]. When patients feel better informed, they experience more person-centered care [1], i.e. care that is respectful of individual preferences, needs and values [2]. Breast cancer patients are, however, increasingly confronted with complex information, as scientific advances continue to reveal the heterogeneity of breast cancer, leading to many subtypes with various treatments options [3, 4]. To make individual, well-balanced, decisions, patients need to be informed not only about the incurability of their disease, but also about the available treatment options and their potential advantages and disadvantages.

Full information-provision is, however, not always achieved; nor may it always be desirable. More specifically, patients are not always explicitly informed about the incurability of their illness [5, 6], or about all available treatment options and potential side effects [5–7]. Doctors sometimes omit the option of no anti-cancer therapy [5–7], and the possibly negative effects of treatment on condition and social functioning [5, 8]. Although the lack of full information-provision can limit informed decision-making, some patients are also reluctant to receive all information [9, 10], especially as their illness progresses [11, 12]. In order to inform patients without overwhelming them, we need to shift our focus beyond current practices and preferences, to a deeper understanding of the challenges oncologists and patients perceive in information-provision (about disease status, available treatment options, and side effects), and possible strategies to address these challenges.

Moreover, specific ways to facilitate information-provision need to be explored. Promising techniques include communication strategies based on placebo-effect principles, such as positive expectations and empathy. Positive expectations can decrease patients’ pain perceptions [13, 14], while empathic behaviors (e.g., reassurance, attentive silence) can decrease anxiety and increase recall [15–19]. The potential of positive expectations and empathy to facilitate information-provision has yet to be explored.

Against this background, the aim of this project is to explore, in the setting of advanced breast cancer, oncologists’ and patients’ views on (i) the challenges of information-provision and (ii) possible strategies to address these challenges, while (iii) exploring the possible facilitating role of positive expectancies and empathy in addressing the challenges. Addressing these aims will help achieve optimal information-provision and person-centered care.

## Methods

### Design

Qualitative study, using semi-structured interviews with breast cancer patients and oncologists.

### Ethical considerations

All study-procedures were submitted to the Medical Ethical Committee of the Netherlands Cancer Institute (NKI-AVL), which exempted the study from formal ethical approval (P18LVW). In addition, the Ethical Committee of Leiden University, Psychology Department, approved the conduct of the study. The participating hospital (St Antonius) approved recruitment in their hospital (Z18.031).

### Sample

#### Patients

Female patients (> 18 years) with incurable breast cancer; sufficient command of Dutch language; cognitively able to provide consent and be interviewed.

#### Oncologists

Providing care for women with advanced breast cancer in a Dutch hospital.

### Recruitment and procedures

#### Patients

We followed principles of purposeful sampling, aiming a variety in participants’ age, disease characteristics, geographical location, education, cultural background.

Participants were recruited – June-Sept 2019 – through the Dutch breast cancer patient advocacy organization and a patient organization for migrant women, involved patient-representatives, snowballing procedures. Via personal contacts and oncologists within the participating hospital we purposefully recruited patients with a non-Western migrant background and/or low educational levels. Patients contacted the research team, who provided more information via telephone and checked inclusion criteria. An information letter/consent form and questionnaire were sent, and interviews were scheduled at patients’ homes or at Leiden University. Written consent was obtained pre-interview and the questionnaire collected/completed. Patients were informed they could always stop the interview.

#### Oncologists

Recruitment occurred via personalized emails, or – for one hospital – via a medical psychologist. Oncologists interested in participating contacted the research team (LV), who provided more information (via email). The information letter/consent form, and questionnaire were sent and the interview was scheduled at the oncologist’s hospital or by telephone. Written consent was obtained pre-interview and the questionnaire collected/completed (or sent by post).

Participants were reimbursed only for travel. Transcriptions were offered for comment/corrections.

### Topic list

A topic list was created (Additional file 1), in collaboration with patient-representatives. The topic guide focused on i) the challenges of informing patients about treatment options, aims and side-effects; ii) strategies how these challenges can be overcome; iii) the possible facilitating role of empathy and positive expectations in reducing these challenges.

### Outcomes

#### Patients

Sociodemographic/ disease characteristics were assessed.

#### Oncologists

Sociodemographic and professional characteristics, and self-perceived confidence in discussing disease status (treatment aims), options and benefits/side effects in advanced cancer were assessed (self-created 1–4 scale).

### Data collection

Interviews were held by one – trained/experienced female – researcher (LV – communication/palliative care/psychology background, or MM – communication/psychology background), for many interviews both were present.

The audio-recorded interviews were transcribed verbatim, and personal identifiers removed. Data-analysis was part of a cyclical process of data collection and analysis. Data collection was stopped when data saturation was achieved.

### Analysis process

Analysis was performed using Thematic Analysis [20]. Firstly, two researchers (LV, MM) (re)read the transcripts to familiarize themselves with the interview data and independently wrote a memo of the most striking findings of each interview (step 1). These memos were subsequently compared and discussed, and initial codes were given to relevant interview fragments (step 2). Using further discussion of memos and initial codes, the two researchers generated themes, which were displayed in a draft figure (step 3). At several points, themes and interim analyses were reviewed and discussed with co-authors (backgrounds in psychology/medicine/nursing/communication) (step 4). Gradually, themes were further defined and named, and again discussed with co-authors (step 5). Lastly, the results section was written and the research question answered (step 6). Input was received from co-authors, and the final figure, displaying the main themes identified, was drawn. Steps 2–5 were supported by software program Atlas.ti. COREQ guidelines for qualitative research were followed for reporting.

## Results

### Participants

**Fig. 1:**
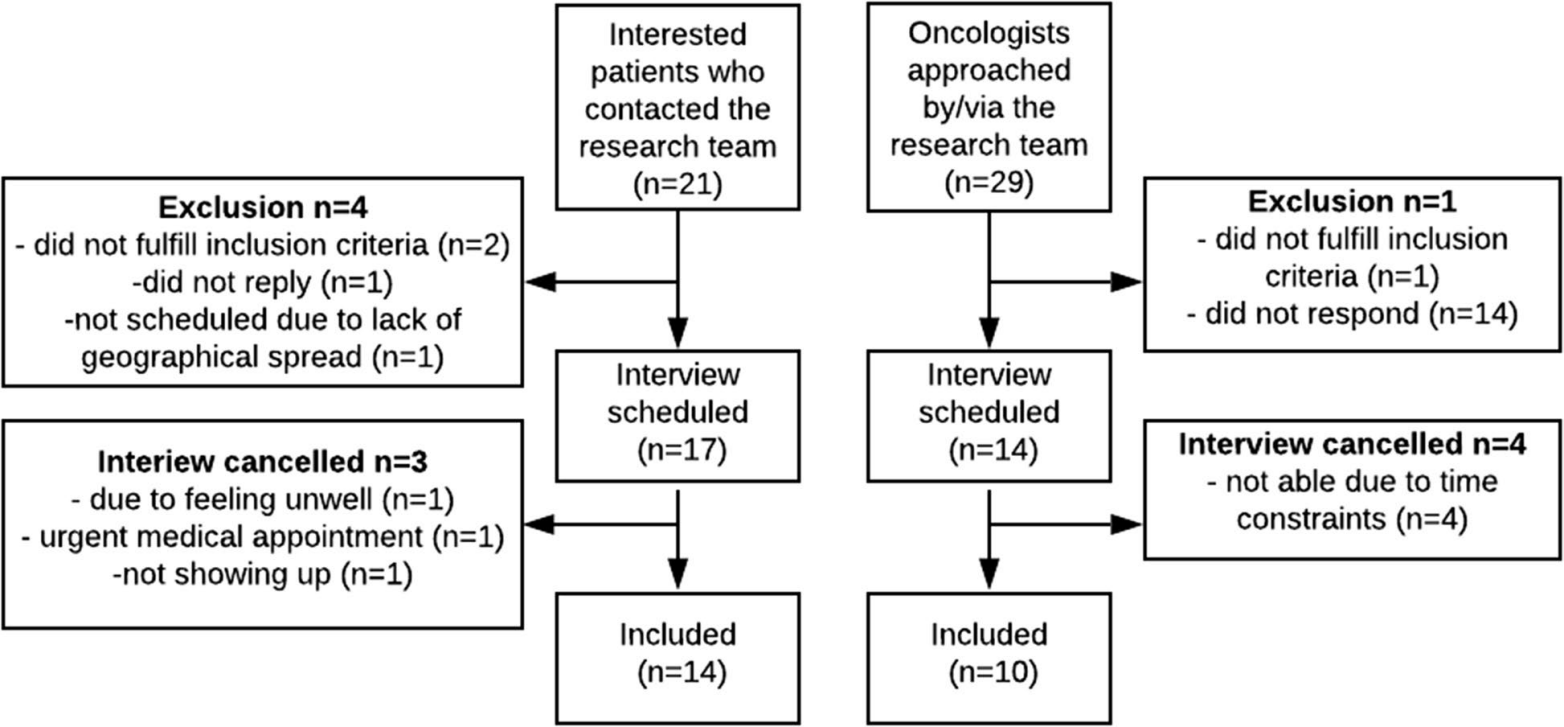
Flowchart of recruitment process of patients and oncologists

Table 1 shows the background characteristics of the 14 patients and 10 oncologists included; Fig. 1 shows the recruitment process.

**Table 1:** Background characteristics of participants

Patients	Total (N = 14)
	M (SD)
**Age**	57.92 (7.40) (Range 46–70)
	N (%)
**Marital status**	
Married (including registered partnership)	7 (50.0)
Single (including divorced, widowed, unmarried)	7 (50.0)
**Highest Education**	
Low (primary education or less)	-
Intermediate-1 (lower education)	5 (35.7)
Intermediate-2 (upper secondary)	2 (14.3)
High (tertiary)	7 (50.0)
**Occupation**	
Paid job	-
Disabled / Sick leave	11 (78.6)
Housewife	-
Retired	2 (14.3)
Voluntary work	1 (7.1)
**Cultural background**	
Dutch	13 (92.9)
Western immigrant	-
Non-Western immigrant	1 (7.1)
**Treatments currently receiving**^**a**^	
Chemotherapy	2 (14.3)
Radiotherapy	-
Hormone therapy	1 (7.1)
Immunotherapy	9 (64.3)
Operation	3 (21.4)
Symptom-oriented treatment (e.g. bone strengthening & rehabilitation)	2 (14.3)
Tumor-oriented treatment possible, but refrained from	4 (28.5)
Tumor-oriented treatment impossible	1 (7.1)
Oncologists	Total (N = 10)
	M (SD)
**Age**	48.50 (7.34) (Range 37–59)
	N (%)
**Sex**	
Male	4 (40)
Female	6 (60)
**Setting**	
Academic	2 (20)
Peripheral	6 (60)
Specialized cancer hospital	2 (20)
**Experience**	
< 5 years	2 (20)
5 – 10 years	2 (20)
> 10 years	6 (60)
**Self-perceived confidence to discuss treatment aims**	
Not at all	-
Moderate	-
Fairly	1 (10)
Very	9 (90)
**Self-perceived confidence to discuss treatment options**	
Not at all	-
Moderate	-
Fairly	1 (10)
Very	9 (90)
**Self-perceived confidence to discuss benefits/side effects**	
Not at all	-
Moderate	-
Fairly	2 (20)
Very	8 (80)

^a^ Women can receive several treatments, so this does not add up to 100%

### Themes

**Fig. 2:**
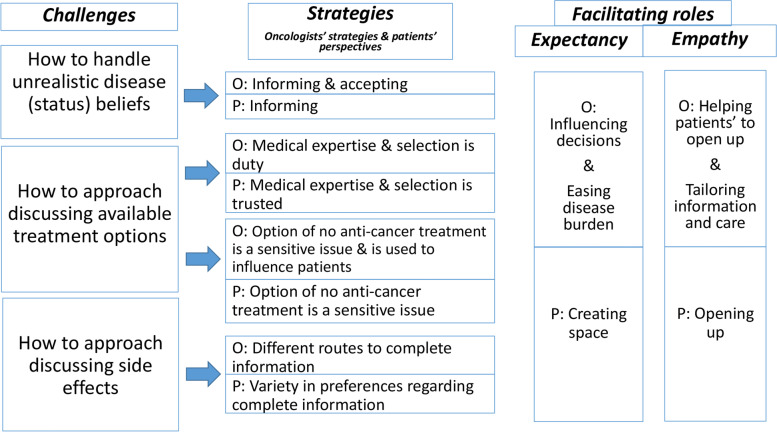
Schematic overview of challenges; oncologists’ used strategies & patient preferences; possible facilitating role of expectancy & empathy

From the analysis of oncologists’ and patients’ data, three main themes were identified, concerning challenges in the discussion of disease status, treatment options, and side effects. Patients often started by mentioning little challenges, mainly focusing on their preferences. Oncologists identified challenges, but considered informing patients a core task. Figure 2 displays themes and possible relations between them.

### Challenge 1: How to handle unrealistic disease (status) beliefs

Oncologists mentioned the challenge of handling patients’ unrealistic disease beliefs. This focused on patients not expecting or accepting the disease’s incurability, and having overly optimistic/pessimistic expectations.

#### Strategy chosen by oncologists: informing & accepting

To address this challenge, oncologists try to provide information – especially concerning the disease’s incurability – and to keep an open dialogue, actively exploring patients’ perceptions. Simultaneously, they seem to accept – temporary – denial of the incurability of the disease. If necessary, e.g. with disease progression or if decision-making is hampered, this may be re-discussed.

*What we often see in clinical practice is that as a coping mechanism people say “I hear you [that the disease is incurable, red], but I don’t want to accept it yet (…). So I’ll say (…) you need to know this to make well-informed decisions, but we don’t need to keep discussing that you’re dying. (4005).*

#### Patients’ preferences: informing

Patients stressed the importance of being clearly informed about their disease status; the incurable status is devastating but essential to know.

*You’ve got two children; so you don’t want to hear it [that the disease is incurable, red]; nobody wants to hear that. But well [it needs to be said clearly, ed]; they cannot keep avoiding it. (3013).*

### Challenge 2: How to approach discussing available treatment options

A second challenge involves discussing treatments options, specifically whether to discuss all options or a selection, and whether to include the option of no anti-cancer therapy.

#### Strategy 1 chosen by oncologists: medical expertise and selection is duty

Oncologists, though acknowledging patients’ right to know all options, seemed to agree that medical expertise and selection is needed. They often select the most effective options (in terms of e.g. efficacy, limited side effects); some select options that differ (e.g. in terms of side effects or hospital visits needed); some actively enquire about patients’ priorities/expectations.

*I generally make a selection. I think most people benefit from your advice; that’s why they’re there. I generally discuss the most effective treatment (…). If there are two equal options, then you discuss the pros and cons of the different options. (ID 4028).*

#### Patients’ preferences: medical expertise and selection is trusted

Patients often expressed faith in oncologists’ medical expertise and appreciated them selecting treatment options. Most did not want to carry sole responsibility for decision-making, arguing that they lacked medical expertise.

*I don’t know about all the treatment options there are so I trust that the oncologist will give me the best treatment (…). He will discuss it with the team; he confers with other oncologists and doctors. That’ll lead to a specific treatment option, and I assume he knows what he’s doing. ( 3034).*

Still, some women were keen to make their own decisions and/or wanted an oncologist to respect their decisions.

*You can also have chemo in tablet form, but because I already wasn’t responding well to it, I said I’m not doing chemo. And she didn’t try to persuade me, which I appreciated. (3025).*

#### Strategy 2 chosen by oncologists: option of no anti-cancer treatment is a sensitive issue & is used to influence patients

The discussion of ‘no anti-cancer treatment’ was a sensitive issue. Some oncologists always mention it, as it is a realistic option. Others acknowledged that it was not always a logical option to discuss, especially for young patients with hormone-sensitive tumors. The option of no anti-cancer treatment was explicitly discussed where the burdens of anti-cancer treatment might outweigh the gains (e.g., with older patients, limited prognosis, sole option of chemotherapy). Introducing the option (or not introducing it) was used to influence patients’ decision-making.

*For patients with hormone-sensitive tumors, you can usually find a treatment with relatively few side effects. Then it’s less obvious to me to suggest the option of no anti-cancer treatment. So I don’t know if I always mention it (…). But I do if I have a treatment in mind that won’t be symptom-free (…), or if the costs and benefits could be out of balance. (4009).*

#### Patients’ preferences: option of no-anti-cancer treatment is a sensitive issue

Patients’ – sometimes strongly expressed – views varied on whether this option should be discussed. For some it was a realistic option that should always be mentioned; for others it equaled the end of available treatments. For most, this option was not relevant at that time. They knew, however, that it was an option, which should be discussed in detail later in their disease trajectory (e.g. when fewer options are available, or quality of life becomes impaired).

*I do think we should have a choice. Those drugs have a lot of side effects (…), so it has to be a choice (3042).*

*We didn’t discuss it [the option of no anti-cancer treatment, ed.], because I had already said that wasn’t an option. I want to continue as long as possible. (3010).*

### Challenge: How to approach discussing side effects

A last challenge is how to discuss possible side effects.

#### Strategy chosen by oncologists: different routes to complete information

Oncologists described several approaches, but ultimately favored full side-effect insight. Most oncologists mentioned common and alarming side effects (more would be overwhelming); a few mentioned all possible side effects (to inform the patient fully and protect against later complaints); some always mentioned specific side effects (e.g. nausea). Oncologists considered it important for patients ultimately to be fully informed – possibly through specialized nurses/written information – to make well-informed decisions. The impact of treatments on patients’ daily functioning was also sometimes mentioned, but patients’ priorities were seldom explored.

*We try to mention the most important things. In the case of chemotherapy, they also talk to a nurse who informs them about almost all possible side effects, and often provides the information on paper as well. (4007).*

*And I always add a bit about what I often see. And how hard people usually find it (…). And I always discuss the important things, like when they should contact us, or dangerous side effects (…). When it comes to choosing treatment A or B, I also discuss the risk of side effects that will really impact quality of life. (4025).*

#### Patients’ preferences: variety in preferences regarding complete information

Patients’ view on the ‘right’ approach for side-effect information also varied. Most patients wanted to be informed of alarming side effects. Preferences varied about whether all side effects should be mentioned, or the most common, personally relevant, or only a limited number. The opportunity to contact the hospital was important; written information and concrete advice on handling side effects was seen as less essential. Most patients were reluctant to receive statistical side-effect information which might not apply to the individual.

*I think they should provide clear guidelines for when it is red alert (…). If you experience this, call immediately and go straight to hospital (3020).*

*If they had given me the whole list of what could happen I would have never started it, and in hindsight I’m glad I did. (3051).*

*That’s why I say: every person responds differently (…). I um, no I don’t really believe in numbers. (3034).*

### The facilitating role of expectancy

When introducing the topic of positive expectations, patients and oncologists stressed the importance of realism. Still, positive expectations can facilitate addressing the challenges encountered, by influencing decision-making, easing patients’ disease burden, and creating space.

#### Influencing decisions: oncologists

Oncologists spoke about how they use positivity to influence treatment decision-making by highlighting benefits, (dis)advantages, and side effects. For example, potential positive outcomes are stressed early in the disease trajectory, while disadvantages are highlighted when oncologists think it might be better to stop intensive treatments.

*If you feel you have little faith in the treatment, you’re perhaps more inclined to highlight how hard it is, whereas if you feel it’s a very important treatment, you indicate more that it’s not that bad. (4018).*

#### Easing disease burden: oncologists

Some oncologists acknowledged that positive expectations can influence how patients cope and experience symptoms and side effects. Positivity was sometimes explicitly used to motivate patients, e.g. to start/continue treatments.

*If people are nauseous from chemo, and I give them a remedy, I’ll add that it works really well. Because I know this can give just that extra placebo effect that stops them feeling nauseous. (4009).*

*Especially chemotherapy – many patients really dread it. So I explain that there are many sorts of chemo, and this one really isn’t so bad usually. (4025).*

#### Creating space: oncologists

Oncologists seldom explicitly mention that positive expectations can create space, but focus on balancing between discussing potential positive and negative outcomes/side effects.

*So I talk about side effects, but immediately add that almost nobody has all side effects, and it varies – from people with almost none to people who do experience many side effects. (4039).*

#### Creating space: Patients

Patients focused on how positive expectations can create space. They often mentioned the importance of receiving information about possible positive treatment outcomes and limited side effects. For some this needed to be balanced against potential negative outcomes (more realistic); others preferred a focus on positivity (something to aim for); some acknowledged they realized themselves things might not go well.

*…that they inform you it may not have much effect, or may not agree with you; but possibly you will respond very well; we just have to try it out, because it differs per person. (3025).*

*Maybe a doctor can help at that point. Maybe it would work, (…). Without fibbing. Just saying ‘it’s possible’. Who knows, let’s just try. (3028).*

Hearing about possible anti-cancer treatment options was described as ‘positive’, especially knowing that there are options available if the present treatment fails.

*Say the tumors start growing, or the blood levels aren’t good, or whatever… that there are still drugs they can try. This drug isn’t the end of the road. And I like that she keeps repeating that, let’s say. (…) That [creating space, ed.] is really important for me. (3042).*

### The facilitating role of empathy

Oncologists see empathy (see Table 2 for the faces of empathy) as the basis for their communication, while acknowledging that it depends on the patient, situation, and doctor-patient relationship.

*It is easier to feel empathy with someone you find likable than someone who actually evokes irritation. (4018).*

**Table 2:** Different faces of empathy

**Oncologists**
Oncologists did not always find it easy to cite concrete examples of how they demonstrate empathy. Still, they did sometimes mention examples of empathic behavior. Firstly, they could think things through with the patient, but from a medical perspective (e.g., suggesting that a daughter get married earlier so her mother could still attend). Secondly, they could reassure the patient that they would not be abandoned (e.g., telling patients that the medical team would do their utmost). Thirdly, they could state how sorry they feel for patients
*And in that way you can give some kind of positive turn to giving that really bad news—because I’d known her for some time, you see. (…) How was it [the wedding, ed.]? It was great. And now she’s going downhill, but she is very grateful to me for saying she should move the wedding forward. (4034)*
*It’s bad enough having to explain to someone that he is incurable ill, but if you say (…) we’re there for you to tackle problems and answer questions, in short, to support you and help you, that does help a bit. I think people do feel a lot better when they go through that door. (4012)*
*And certainly if there is anguish, I really feel for them, and I express in words that I empathize with them. (4039)*
**Patients**
Patients spoke in detail about the different forms of empathy oncologists could express, based on realistic foundations. Firstly, they highly appreciated the oncologist thinking things through with them from a medical and a practical perspective: for example, enquiring about the patient’s home situation and whether they need practical help, or trying to think about the best treatments in line with the patient’s preferences
*Right at the start our oncologist asked why we didn’t have home help. (…) So the fact that a doctor comes back to it like that, and says ‘think about it – you’re entitled to it’ (…) – that’s really great. But that’s what empathy is – thinking with the other person and imagining what you would feel yourself. (3018)*
Secondly, they appreciated it if the doctor showed an interest in the patient and took them seriously, for example sincerely enquiring how patients and their loved ones were doing, asking about specific events such as holidays, considering patients’ thoughts, preferences, or symptoms seriously
*We went to see him, and just after the diagnosis my husband got a tattoo, and he noticed. One time he was in the middle of a conversation, then he looks at my husband, and says “hey”, “you didn’t have that tattoo last time”, he says. And those are the kinds of things I think – oh, he sees. And I just like that about him – it’s genuine, and it shows you’re really interested. (3034)*
*And interest, and takes you seriously, and listens to what you say. Because that is, well, that’s always really important. That you have the feeling the doctor really wants to know how you are and what you think about something. (3018*)
Thirdly, patients appreciated it if oncologists took enough time for conversations – especially difficult ones –, or at least gave the impression of having time and expressed a sense of calm
*Even if she’s running an hour late, that she still gives you the feeling she has time for you. And that you can ask whatever you want. (…) Yes, she radiates a sense of calm, I feel (3042)*
Lastly, they did not seem to expect oncologists to provide specific psychological assistance, which they sought from other sources, such as specialized nurses, psychologists, or fellow patients

*Of course it has to do with my illness, but I’d rather he [the oncologist, ed.] focused on my treatment and everything related to it. I’ll find other help for the psychological side of things. (3010)*

Patients see expressions of empathy as important, but with a firm medical basis: the oncologists’ medical expertise is paramount. A lack of empathy was perceived as hurtful.

*Let’s say [the medical part, ed.] 75%, and the personal part 25%; asking how I’m doing, how things are at home (…). And it’s not strictly necessary, because basically I’m there for my disease and not to make friends. (3034).*

However, although this is not mentioned as explicitly as for expectancy, oncologists’ expressions of empathy may facilitate the process of addressing the challenges encountered, by helping patients to open up (e.g. about their thoughts, feelings, priorities, personal situation), thus allowing information and care to be tailored to their needs.

#### Helping patients to open up and tailoring information and care: oncologists

Some oncologists mentioned that expressions of empathy can lead patients to open up and confide their priorities. This in turn may facilitate the provision of person-centered information and – treatment – care.

*Because the better you think you understand the other, the more there’s a click, let’s say – that means you understand one another better and you can get closer to the core of those people, what motivates that person? And what is important to that person? Yes, I do believe the better you can unearth that, the more carefully you can advise them. (4005).*

#### Opening up: patients

A few patients also stated that if an oncologist shows empathy, they are more willing to open up and share their feelings or story.

*You have to feel comfortable with someone; you’re not going to reveal yourself to just anybody. If there’s some asshole of a surgeon opposite me, then I’m like whatever. Yes, you have to have someone you feel comfortable with. (3002).*

## Discussion

This study aimed to explore oncologists’ and patients’ views on challenges of providing information to women with advanced breast cancer, and possible strategies to address these challenges, meanwhile exploring the possible facilitating role of positive expectations and empathy, with the ultimate aim to come to patient-centered care. Integrating oncologists’ and patients’ views, we observed that oncologists face challenges regarding handling unrealistic beliefs, and regarding choosing how to discuss treatment options (including no anti-cancer therapy) and possible side effects. Strategies to address these challenges include balancing information-provision with acceptance of denial, and using medical expertise to guide treatment discussions (though discussing the option of no anti-cancer therapy remains a sensitive issue). A variety of approaches and preferences for discussing side effects exists. Positive expectations and empathy can facilitate information-provision by – amongst others – creating space and helping patients to open up.

In general, oncologists reported more challenges in information-provision than patients. While patients are increasingly involved in agenda-setting [21], oncologists bear the responsibility for explaining complex medical information in a manner patients can understand [22]. This imbalance sheds light on the challenges and also strategies being formulated from the oncologists’ viewpoints while they derived from both oncologists’ and patients’ data.

A first main challenge that arises in information-provision is handling patients’ unrealistic disease beliefs, mainly about the diseases incurability. While patients stressed the importance of realism, oncologists also leaned towards accepting denial. Previous studies have found that patients place great importance on realism [23–25]. Simultaneously, denial of a life-threatening diagnosis occurs in 4–47% of patients, can be temporary [26], and can serve as an adaptive coping mechanism [27]. The strategy endorsed by clinicians in our study (and beyond [28]) – accepting temporary denial after providing realistic information – may be the best way to approach unrealistic beliefs.

A second core challenge is which treatment options to discuss: all or a selection. Patients and oncologists both mentioned that medical expertise is essential for selecting treatment options, while individuals held strong views on whether the option of no anti-cancer therapy should be mentioned. Previous research has found that oncologists want to discuss “best options” [29] – not always including no-treatment [7, 30] – while patients have faith in oncologists’ treatment recommendations [12, 29, 31–33]. This seems to contradict legal requirements [34–36] and guidelines stressing the importance of mentioning all available treatment options [37], including solely optimal supportive care [22]. Perhaps such guidelines overlook patients’ need for medical guidance, and the emotional impact of raising the option of no anti-cancer therapy. In an era of increased focus on patients’ responsibility, we should not overlook the sensitivity of discussing no anti-cancer treatment, and the importance of medical expertise in guiding patients through difficult, uncertain times.

A last challenge, with no clear solution, seems to be how to discuss potential side effects. Patients and oncologists agreed that alarming attention-requiring side effects should be discussed; however, while patients varied in their preferences regarding additional information on side effects, oncologists favored complete information-provision. Given the ethical and legal requirements for “informed consent” [35–37] to be based on full information and insight, oncologists’ perspectives are understandable. Some previous studies have found patients wishing to receive more [1, 38] and not too much [33] information on side effects. Our study suggests that above all information on serious side effects is appreciated. Whether mild/nonspecific side effects should also always be discussed remains unanswered. Moreover, the variety in patients’ preferences regarding the discussion of side-effects (and treatment options), once again highlights the importance of adjusting information to each individual patients’ needs and preferences, in order to come to patient-centered care. In order to tailor information, oncologists have been advised to ask patients about their information-preferences [39].

Next, we also explored the roles of positive expectations and empathy in facilitating information-provision. For patients, positive expectations might provide hope, as stressing potential positive outcomes and treatment options has previously been described as hopeful [12, 24, 25]. For oncologists, who seem regularly to mention possible positive treatment outcomes [40], stressing potential advantages or disadvantages can serve as a tool to influence decision-making, as also previously found [30]. In this earlier study, oncologists acknowledged to sometimes provide information regarding hams and benefits in line with their preferred (treatment) option. It remains to be determined whether, as suggested in our study, positive expectations can ease disease burden in advanced cancer; positive effects on non-cancer symptoms have been described [13, 14] while it might also improve coping and thereby symptom burden [41].

Where empathy is concerned, some oncologists and patients agreed it can prompt patients to share personal preferences and information, which oncologists can use to tailor information and care plans. In this regard, we could speculate that the trust patients placed in oncologists’ treatment plans may also have originated from empathy. Relationship-building and information-provision are often seen as distinct elements of the medical encounter [42, 43], but our study seems to question this. This view is supported by previous findings demonstrating that empathy can facilitate information-processing and improve patients’ recall [16–18]. Providing empathy need not be time-consuming [44, 45], with studies demonstrating that effective empathy can be achieved in less than 40 s [16, 44]. It is a powerful, and often under-used [45], tool oncologists can use to make patients feel better [46, 47] and optimize information and care.

Our study has strengths and limitations. strengths mainly concern the often overlooked focus on challenges in information-provision and the dual focus on both patients’ and oncologists’ perspectives. Limitations mainly concern the generalizability of results beyond our study sample. Despite various attempts to include women with nonwestern migrant backgrounds and/or low educational level, mainly high-educated Caucasian women participated. Second, oncologists with a specific interest in communication might have participated, biasing our results.

Future studies might focus on how to tailor and match individual patients’ preferences – specifically on discussing the option of no anti-cancer treatment – with oncologists’ information-provision approaches. Assessing patients’ information needs pre-consultation, as one oncologist suggested, might help. More insight is needed into how to inform patients about potential side effects in a way that is tailored to individuals’ preferences while adhering to information-provision obligations, and without eliciting nocebo effects. In addition, we encourage future studies to be more culturally and health literate diverse, and to include males and other cancer types, as these variables might influence communication needs [48–51].

## Conclusions

Integrating patients’ and oncologists’ views, oncologists can provide realistic information while also – temporarily – accepting denial, and can use their medical expertise when addressing challenges around unrealistic beliefs and the discussion of treatment options. Finding ways to tailor discussions of no anti-cancer treatment and side-effect information are needed. Positive expectations and empathy might facilitate – tailored – information-provision, leading ultimately to patient-centered care at the heart of medicine.

## Supplementary Information

**Additional file 1.**

## Data Availability

The datasets generated and/or analysed during the current study are not publicly available due to the qualitative nature of the study. We encourage individual researchers interest in our data to contact us directly (Liesbeth van Vliet, via l.m.van.vliet@fsw.leidenuniv.nl).
