# Supplementary material for: Addressing challenges in information-provision: a qualitative study among oncologists and women with advanced breast cancer
Source: BMC Palliat Care. 2021 Sep 14;20:142. doi: 10.1186/s12904-021-00836-w (PMC8442372; doi:10.1186/s12904-021-00836-w)
Supplement: Supplementary file 1 — Additional file 1. [file 12904_2021_836_MOESM1_ESM.docx]

Additional file 1 - (Abbreviated) Topic list for interviewing patients and oncologists

**Topic list for oncologists**

***What are the challenges in informing patients about treatment options, aims (disease status, red) and side effects (challenges)?***

***How can these challenges be overcome/met (strategies)?***

***Can the use of empathy and positive expectations reduce these challenges (facilitation)?***

**Topic list for patients**

***What are the challenges in discussing treatment options, aims (disease status, red) and side effects (challenges)?***

***How can these challenges be overcome/met (strategies)?***

***Can the use of empathy and positive expectations reduce these challenges (facilitation)?***
